# A Multicenter Observational Study Comparing Survival of Pugs and Dogs of Other Breeds With Protein‐Losing Enteropathy

**DOI:** 10.1111/jvim.70100

**Published:** 2025-05-15

**Authors:** Harry Swales, Daniel J. Batchelor, Tereza Bodnárová, Andrew Kent, Susan Campbell, Adam G. Gow, Margaux Kuijlaars, Peter‐John M. Noble, Anna Threlfall, Paolo Silvestrini, Jennifer Stallwood, Harry Warwick, Alexander J. German

**Affiliations:** ^1^ Department of Internal Medicine Bridge Referrals Boldon Colliery UK; ^2^ Small Animal Teaching Hospital University of Liverpool Neston UK; ^3^ Willows Veterinary Centre & Referral Service Solihull UK; ^4^ Blaise Veterinary Referral Hospital Birmingham UK; ^5^ University of Edinburgh, The Royal (Dick) School of Veterinary Studies and the Roslin Institute, Hospital for Small Animals Midlothian UK; ^6^ Small Animal Hospital School of Veterinary Medicine, University of Glasgow Glasgow UK; ^7^ Davies Veterinary Specialists Higham Gobion UK; ^8^ Ryan Veterinary Hospital University of Pennsylvania Philadelphia Pennsylvania USA; ^9^ Bristol Veterinary School University of Bristol Bristol UK; ^10^ Bristol Vet Specialists Bristol UK; ^11^ Northwest Veterinary Specialists Cheshire UK; ^12^ Institute of Life Course and Medical Sciences, University of Liverpool Neston UK

**Keywords:** chronic enteropathy, chronic inflammatory enteropathy, diarrhea, enteropathy, inflammatory bowel disease, lymphangiectasia, PLE

## Abstract

**Background:**

Protein‐losing enteropathy (PLE) in dogs often carries a guarded prognosis, and it is unclear if survival differs among breeds.

**Hypothesis/Objectives:**

Survival of pugs with PLE is shorter than that of other breeds of dogs with PLE.

**Animals:**

Forty‐seven pugs and 148 dogs of other breeds were diagnosed with PLE at seven United Kingdom (UK) referral hospitals.

**Methods:**

Retrospective, multicenter observational study. Case records were reviewed to identify dogs diagnosed with PLE. Cox's proportional hazards regression was used to determine variables associated with survival.

**Results:**

Median (interquartile range) survival in pugs with PLE and dogs of other breeds was 104 (22–719) days and 759 (61–1632) days, respectively (*p* = 0.002). The hazard of death was higher in pugs (hazard ratio [HR]: 1.961; 95% confidence interval [CI]: 1.108–3.741; *p* = 0.002) than in other dogs. Neutrophil counts in peripheral blood were associated with an increased hazard of death (HR change per 1 × 10^9^/L: 1.045; 95% CI: 1.014–1.077; *p* = 0.004), whereas cobalamin concentration (HR: 0.995; 95% CI: 0.991–0.999) and cobalamin supplementation (HR: 0.517; 95% CI: 0.271–0.988) were positively associated with decreased hazard of death. A time‐dependent effect on survival was identified for serum globulin concentrations, whereby globulin concentration was positively associated with hazard of death in dogs surviving 61–959 days (HR: 1.126; 95% CI: 1.040–1.219) and > 959 days (1.253; 95% CI: 1.048–1.497), but not 0–60 days (HR: 0.949; 95% CI: 0.891–1.011 days).

**Conclusions and Clinical Importance:**

Results of our observational study suggest a worse prognosis for pugs with PLE compared to a selection of dogs of other breeds seen at UK referral centers.

**Abbreviations**
CCECAI: Canine Chronic Enteropathy Clinical Activity Index
CE: chronic enteropathy
CHOP: cyclophosphamide, hydroxydaunorubicin, vincristine, prednisolone (multi‐drug chemotherapy protocol)
CI: confidence interval
CIBDAI: Canine Inflammatory Bowel Disease Activity Index
HR: hazard ratio
IQR: interquartile range
MST: median survival time
PLE: protein‐losing enteropathy
PLN: protein‐losing nephropathy
RI: reference interval
VIF: variance inflation factor

## 1 Introduction

Protein‐losing enteropathy (PLE) is a syndrome in dogs that typically causes chronic diarrhea and is thought to be caused by chronic enteropathies (CE), such as primary lymphangiectasia, small or large cell lymphoma, and idiopathic chronic inflammatory enteropathy. However, it is not yet clear whether PLE is simply a severe presentation of CE or a separate disease entity [1].

There is a clinical impression that dogs of the pug breed respond less favorably to treatment for PLE in comparison to other breeds of dog, whereby an initial therapeutic response is quickly followed by relapse within 2–3 months, with no response to subsequent rescue treatment. However, despite these observations, there have been few scientific studies examining differences in response to PLE among various breeds. Based on the hypothesis that clinical response is worse in pugs with PLE than in other breeds, the aim of our study was to compare survival between pugs and a control group of dogs of different breeds diagnosed with PLE at United Kingdom (UK) referral centers.

## 2 Materials and Methods

### 2.1 Study Design and Ethical Considerations

Ours was a multicenter, retrospective study involving seven UK veterinary referral hospitals: Langford Vets, University of Bristol (UoB); Davies Veterinary Specialists (DVS); The Royal (Dick) School of Veterinary Studies Hospital for Small Animals, The University of Edinburgh (UoE); Small Animal Hospital, University of Glasgow (UoG); The Small Animal Teaching Hospital, University of Liverpool (UoL); Northwest Veterinary Specialists (NWS); and Willows Veterinary Centre & Referral Service (WVCR). At all hospitals, cases were seen by a Diplomate of the European or American Colleges of Veterinary Internal Medicine (ECVIM or ACVIM), or by a resident in an ECVIM training program working under the supervision of a Diplomate. During the initial phase (2016–2018), five hospitals were recruited, with an additional two hospitals being recruited in 2019. The census dates for data collection at each hospital were as follows: August 2018 (UoB), April 2018 (DVS), February 2018 (UoE), November 2018 (UoG), October 2018 (UoL), November 2019 (NWS), and August 2019 (WVCR). At each hospital, electronic patient records were searched for all dogs diagnosed with PLE between January 2011 and the respective census date.

Protein‐losing enteropathy was defined as any gastrointestinal condition with a concurrent serum albumin concentration less than the reference interval of the clinical pathology laboratory used by the respective referral hospital, where a non‐gastrointestinal cause (e.g., hepatic disease, protein‐losing nephropathy, and blood loss) was not evident. The University of Liverpool Veterinary Research Ethics Committee reviewed and approved both the animal welfare and research ethics aspects of the project (VREC500ab). Owners gave written permission for anonymized animal data to be included in their search.

### 2.2 Eligibility Criteria

Dogs were eligible for inclusion if their illness fitted the definition of PLE described above, and the following information was available in their electronic patient records: breed, serum albumin concentration at the time of initial presentation, date of diagnosis, and date of either last available follow‐up or death (including euthanasia). Where the diagnosis was unclear, cases were evaluated by a subgroup of authors (HS, DB, PN, PS, and AG), and a decision was made by consensus. Owing to case‐identifier blinding, it was not possible to identify individual cases, and no attempt was made to exclude cases that contributed to two previous studies from the UoL [2] and UoE [3].

All dogs of the pug breed, and a random selection of dogs of other breeds seen over the same time period, were included. Given difficulties in obtaining consent for data inclusion from dogs of other breeds at one hospital (DVS), controls were only recruited from six hospitals. At each hospital, dogs of other breeds diagnosed with PLE during the study period were sequentially assigned a study number (from 1 to *n*, where *n* was the number of dogs of other breeds from that hospital). The “RANDBETWEEN” function of an electronic spreadsheet software (Excel version 1086, Microsoft Inc., Washington) was used to produce a sequence of 35 random numbers for each hospital, enabling at least 20 dogs of other breeds to be selected for each hospital. Given that two centers (WVCR and NWS) were enrolled at a later stage, an unequal number of controls were recruited from each hospital, with four hospitals contributing 25–35 controls (UoB, UoE, UoG, and UoL) each and two hospitals (NWS and WVCR) contributing 15–20 controls.

### 2.3 Data Collection

For each case, details of signalment, clinical pathology, histopathology, and treatment were recorded. Signalment data included age, sex, neuter status, and weight at the time of diagnosis. Laboratory data collected included total protein (g/dL), albumin (g/dL), and globulin (g/dL) concentrations, neutrophil count (×10^9^/L), lymphocyte count (×10^9^/L), and serum cobalamin concentration (ng/L). Samples were analyzed by different laboratories and with a range of different analyzers, and thus laboratory‐specific reference intervals (RIs) were used. When available, the results of histopathological analysis of intestinal biopsy specimens were categorized based on the predominant inflammatory or neoplastic histopathological diagnosis. The preferred diagnostic pathology service of each hospital conducted the histopathological examination. However, biopsy samples were examined by diplomates of the American (ACVP) or European (ECVP) Colleges of Veterinary Pathology, Fellows of the Royal College of Pathologists (FRCPath), or by residents in an ECVP‐training program working under the supervision of a diplomate. Information on whether biopsy material was collected endoscopically or surgically was not recorded. Diagnosis of small‐cell alimentary lymphoma and intermediate to large‐cell alimentary lymphoma was predominantly based upon histopathology, although immunohistochemistry or clonality assays were performed if requested by the attending veterinarian.

### 2.4 Details of Treatment

Details about diets used in case management were recorded and then categorized according to their predominant characteristic, such as hydrolyzed, low fat, or other (including single‐source protein, highly digestible, and home‐prepared diets). Dogs were categorized as having “no diet recommendation” when the attending veterinarian had not specified a particular diet to use.

Details of all drugs used for each case were recorded, including immunosuppressive and adjunctive treatments. “Initial immunosuppressive treatment” was defined as the first immunosuppressive agent(s) used after diagnosis, and “all immunosuppressive treatment” encompassed all immunosuppressive agents used at any time during follow‐up. Dosages of specific immunosuppressive agents were not recorded. The “no immunosuppressive treatment” category encompassed dogs that responded to dietary treatment alone or were not given any immunosuppressive drug before their death or euthanasia. Use of adjunctive treatments was recorded, including antimicrobials (e.g., metronidazole and tylosin), anti‐thrombotics (e.g., aspirin and clopidogrel), anthelmintics (e.g., fenbendazole), or cobalamin supplementation (enteral or parenteral).

### 2.5 Classification of Response to Treatment

The response to the initial treatment and any treatment used were categorized as no response, partial response, or complete response based on the methodology of a previous study [4]. Dogs were classified as “no response” when the serum albumin concentration did not increase to > 1.5 g/dL or, if already > 1.5 g/dL, did not increase by > 0.1 g/dL after treatment; dogs were classified as “partial response” when the albumin concentration increased to 1.6–2.2 g/dL or, if already between 1.6 and 2.2 g/dL, increased by > 0.1 g/dL, but remained less than the lower limit of the respective hospital laboratory's RI; dogs were classified as “complete response” when the serum albumin concentration returned to within the respective hospital's laboratory's RI after treatment. The initial response to treatment was defined as the best response category achieved after the initial recommended treatment, and before other treatments were added, whereas response to “any” treatment was defined as the best response category achieved at any point during treatment.

Survival data were acquired from details recorded in the electronic patient records or, where necessary, by telephone communication with the referring veterinarian or owner. For ethical reasons, only a single attempt was made to communicate with the owner. The status of all dogs reported to be alive was confirmed to be accurate within 1 month of the census date at each hospital. Duration of follow‐up was calculated from the date of the first presentation to either the date of death or euthanasia or the date of the last available follow‐up for dogs that were still alive.

### 2.6 Statistical Analysis

An a priori sample size calculation was performed using statistical software (G*Power version 3.1.9.2, G*power Team, Düsseldorf, Germany), with expected clinical response based on survival data from the preliminary results of a recent study evaluating dogs with CE [5]. Assuming an 80% power, an *α* of 0.05, and a 1:1 ratio between pugs and dogs of other breeds, an estimated sample size of 70 dogs would be necessary to detect a 33% difference in survival.

Data from all hospitals were entered into a single electronic spreadsheet (Excel version 1086). Signalment data were available in at least 185/195 (95%) dogs. Total protein, albumin, and globulin concentrations were available in 195/195 (100%) of dogs per the inclusion criteria. Serum cobalamin concentration was available in 153/195 (78%) dogs, and neutrophil and lymphocyte counts were available in 191/195 (98%) dogs. Intestinal biopsy results were available in 155/195 (79%) dogs, including 32 pugs and 123 dogs of other breeds. Dietary recommendations were made in 173/195 (89%) dogs. Whether or not immunosuppressive medication was recommended at initial presentation was available in 195/195 (100%) of dogs. Three dogs (two pugs and one other breed) were excluded because of inability to verify follow‐up data within 1 month of data collection. Response‐to‐treatment data were available in 137/148 dogs (93%). Free‐text comments on the suspected cause of death were available in 97/116 (84%) dogs that died. Continuous data are reported as median (interquartile range [IQR]), whereas categorical data are reported as proportions with the corresponding percentage in brackets.

Statistical analysis was performed using an online open‐access statistical software program and environment (R, version 4.4.1 [6]) with several additional packages as follows: “car” version 3.1.2 [7], “dlookr” version 0.6.3 [8], “dplyr” version 1.1.4 [9], “effectsize” version 0.8.7 [10], “ggplot2” version 3.5.1 [11], “ggsurvfit” version 1.1.0 [12], “Hmisc” version 5.1.2 [13], “readxl” version 1.4.3 [14], “rms” version 6.8.1 [15], “survival” version 3.6‐4 [16], “survminer” version 0.4.9 [17], and “vtable” version 1.4.6 [18]. All *p* values were two‐sided, and significance was set at < 0.05. For the purposes of statistical analysis, ages < 1 year were rounded to 1 year, whereas laboratory results below the limits of detection were rounded to the reported detection limit. All data sets of continuous data were first tested for normality using the Shapiro–Wilk test and visual assessment of *Q*–*Q* plots. Given that the majority did not follow a normal distribution despite transformation, nonparametric analyses were used for baseline analyses. Continuous variables were analyzed by using the Mann–Whitney test with the effect size determined by calculating the rank biserial, interpreted according to previously described rules [19], with larger values (positive or negative) indicating larger differences between groups (extremely small: < 0.05: very small: 0.05–0.10; small: 0.10–0.20; medium: 0.20–0.30; large: 0.30–0.40; and very large: > 0.40). Categorical variables were analyzed using Fisher's exact test, with Cramer's *V* used to indicate effect size (interpreted as previously described [20]), whereby values of 0.10, 0.30, and 0.50 denoted small, medium, and large effects, respectively. Given that multiple baseline comparisons were made, *p* values were adjusted to control the false discovery rate using the Benjamini–Hochberg procedure [21].

Cox's proportional hazards regression was used to evaluate associations between survival and independent variables with models stratified by center. Survival time was calculated as the time from diagnosis to censoring (the last visit where follow‐up was available) or death (euthanasia or other). Baseline variables tested were age (years), sex, neuter status, weight, and breed (pug vs. other breed). Clinicopathological variables tested were serum protein concentrations (total proteins, albumin, and globulins), serum cobalamin concentration, neutrophil count, and lymphocyte count. These variables were tested both in a continuous and categorical form, with the latter being created using cut points at either the upper or lower limit of the RI. For histopathological analysis, a binary variable was created denoting whether or not histopathological examination of biopsy samples had been performed, whereas a second binary variable was created for the presence of lymphangiectasia. Dietary recommendations were categorized as hydrolyzed, low‐fat, or other diet, with hydrolyzed diet used as the reference category. Separate binary variables were created for the use of corticosteroids (where the dog received any type of corticosteroid) and the use of other immunosuppressive drugs. The latter category encompassed “single agent non‐steroidal immunosuppressive agent,” corticosteroid, and “an additional non‐steroidal immunosuppressive agent,” or “multiple non‐steroidal immunosuppressive agents.”

Initially, simple regression was undertaken with all variables assessed separately, and interactions between variables were assessed where clinically relevant. Continuous variables were tested in a categorical form, a continuous linear form and also with the inclusion of restricted cubic splines (using the rcs function from the “rms” package) to allow the association between the predictor and outcome variable to be nonlinear. The approach that best fitted the data and met model assumptions was used in subsequent analyses. A multiple regression model was built, which initially included all variables that were *p* < 0.05 on either simple regression, or when a clinically relevant interaction was evident. This initial model was first refined by resolving any multicollinearity issues. To do so, variance inflation factors (VIFs) were calculated and, where possible multicollinearity was identified (VIF > 4), and the variable with the highest VIF was removed. Competing models were tested in a backward and forward stepwise fashion, with the addition or removal of variables, and the best‐fit model was chosen using the Akaike information criterion (AIC) and Bayesian information criterion (BIC). With this approach, the model was repeatedly refined with the addition or removal of variables until the model with the smallest AIC and BIC was found.

Possible violations of the proportional hazards assumption were assessed by examining Schoenfeld residuals and testing them statistically using the cox.zph function of the survival package. Any such violations were resolved by adding a time‐dependent interaction to the model, which involved dividing the survival time variable at specified points, using the survSplit function from the “survival” package. Doing so meant that survival was assessed over three separate intervals: 0–60 days (time group 1), 61–959 days (time group 2), and > 959 days (time group 3), with the cut points corresponding to the IQR. Possible outliers were identified by examining a plot of deviance residuals, whereas a plot of DFbetas was used to identify influential observations. The assumption of linearity was visually assessed by examining a plot of Martingale residuals vs. each continuous variable. Results are expressed as hazard ratios (HRs), or adjusted HR, along with the associated 95% confidence interval (95% CI), and *p* value. Kaplan–Meier curves were created to illustrate differences between models included in the final model. Given that there were missing data for some variables (as described above), a sensitivity analysis was conducted to determine their impact on the results. Here, missing data were replaced by multiple imputation using bootstrapping and predictive mean matching. To do so, the “aregImpute” function of the “Hmisc” package was used. The final multiple regression model was repeated using the imputed data set to enable comparison with results obtained using the original data set (Table S1).

## 3 Results

### 3.1 Study Dogs

One hundred ninety‐five dogs were included (UoB, 31; DVS, 5; UoE, 38; UoG, 32; UoL, 42; NWS, 22; and WVCR, 25), comprising 47 pugs and 148 dogs of other breeds (Table 1). Age was not different between pugs and dogs of other breeds (*p* = 0.557), and no differences in sex were found between groups (pugs: female, 24/47, 51%; other breeds: female, 73/148, 49%; *p* = 0.89). Neuter status was not different between groups, with 37/47 (79%) and 119/148 (80%) of dogs being neutered in pugs and dogs of other breeds, respectively (*p* = 0.87). However, not surprisingly, weight on presentation was less in the pug group (7.8 kg; IQR: 6.7–8.7) compared with the non‐pug group (15.5 kg; IQR: 8.5–23.1).

**TABLE 1:** Baseline group characteristics for pugs and other‐breed dogs.

Variable	Pugs	Other‐breed dogs	*p* ^a^	Effect size^b^
Center			0.01	0.28 (Medium)
University of Bristol	7	24		
Davies Veterinary Specialists	5	0		
University of Edinburgh	4	34		
University of Glasgow	7	25		
University of Liverpool	12	30		
Northwest Veterinary Specialists	7	15		
Willows Veterinary Centre and Referral Service	5	20		
Age (years)	7.0 (6.0–8.0)	8.0 (6.0–9.4)	0.56	0.09 (Very small)
Sex (male/female)	24/23	73/75	0.87	0.00 (Tiny)
Neuter status (neutered/entire)	37/10	119/29	0.87	0.00 (Tiny)
Weight (kg)	7.8 (6.8–8.7)	15.5 (8.5–23.1)	< 0.001	0.57 (Very large)
Breeds	Pug (47)	Cocker Spaniel (14); Border collie (13); Labrador retriever (10); Cross breed (9); Jack Russel Terrier (9); Staffordshire Bull Terrier (9); German shepherd (7); Yorkshire terrier (6); Cavalier king Charles spaniel (5); Hungarian Vizsla (3); Pointer (3); Rottweiler (3); Springer spaniel (3);West Highland white terrier (3); Bichon Frise (2); Border terrier (2); Boxer (2); Golden retriever (2); Greyhound (2); Lhasa Apso (2); Miniature Dachshund (2); Shetland sheepdog (2); Wheaten Terrier (2); Whippet (2); Basset Griffon (1); Basset Hound (1); Beagle (1); Belgian shepherd (1); Bernese mountain dog (1); British bulldog (1); Bull Mastiff (1); Cockerpoo (1); Collie non‐specified (1); Corgi (1); Dogue de Bordeaux (1); Doberman (1); English bull terrier (1); English setter (1); Flat coat retriever (1); Fox terrier (1); Glen of Imaal Terrier (1); Italia Greyhound (1); Jackapoo (1); Labradoodle (1); Lakeland terrier (1); Northern Inuit (1); Papillon (1); Parson's terrier (1); Scottish terrier (1); Shar pei (1); Shih Tzu (1); Perro de Agua (1); Tibetan terrier (1); Weimaraner (1); Welsh springer spaniel (1)	—	—

*Note:* Values are median (IQR) for continuous variables and number for categorical variables.

^a^ Comparisons made with Mann–Whitney test (continuous variables) or Fisher's exact test (categorical variables); the false discovery rate was controlled by correcting all *p* values using the Benjamini–Hochberg adjustment [22].

^b^ Effect size used for continuous variables was the rank biserial and according to the rules of Funder and Ozer [23], with larger values (positive or negative) indicating larger differences between groups (tiny: < 0.05: very small: 0.05–0.10; small: 0.10–0.20; medium: 0.20–0.30; large: 0.30–0.40; and very large: > 0.40); effect size used for Fisher's exact test Cramer's *V*, interpreted according to Cohen [24], whereby values of 0.10, 0.30, and 0.50 denoted small, medium, and large effects, respectively.

### 3.2 Clinical Pathology

Clinical pathological data at presentation are summarized in Table 2. Total protein concentration (pugs: 3.2 g/dL; IQR: 2.8–3.9; dogs of other breeds: 3.4 g/dL; IQR: 3.0–4.1; *p* = 0.35), serum albumin (pugs: 1.4 g/dL; IQR: 1.2–1.9; dogs of other breeds: 1.6 g/L; IQR: 1.4–1.8; *p* = 0.10) and globulin concentrations (pugs: 1.9 g/dL; IQR: 1.7–2.4; dogs of other breeds: 1.8 g/dL; IQR: 1.5–2.2; *p* = 0.17) did not differ between groups. However, serum cobalamin concentration was higher in pugs (353 ng/L; IQR: 194–589) compared with the dogs of other breeds (186 ng/L; IQR: 150–368; *p* = 0.02), whereas more dogs of other breeds (60/118, 51%) had a serum cobalamin concentration less than the respective laboratory's RI than did pugs (8/35, 23%; *p* = 0.02).

**TABLE 2:** Baseline clinical pathology findings in pugs and other breeds of dogs with PLE.

Variable	Pugs	Other breeds	*p* ^a^	Effect size^b^
Biochemistry				
Total protein (g/dL)	3.2 (2.8–3.9)	3.4 (3.0–4.1)	0.35	0.09 (Very small)
Albumin (g/dL)	1.4 (1.2–1.9)	1.6 (1.4–1.8)	0.10	0.18 (Small)
Globulin (g/dL)	1.9 (1.6–2.4)	1.8 (1.5–2.2)	0.17	0.15 (Small)
Cobalamin (ng/L)^c^	353 (194–589)	186 (150–368)	0.02	0.30 (Large)
Cobalamin < RI (ng/L)^c^	23%	51%	0.02	0.22 (Medium)
Hematology				
Neutrophil count (×10^9^/L)^c^	14.04 (9.51–19.76)	10.29 (7.10–14.40)	0.02	0.27 (Medium)
Neutrophil count < RI (×10^9^/L)	6%	2%	0.18	0.08 (Very small)
Neutrophil count > RI (×10^9^/L)^c^	62%	43%	0.06	0.15 (Small)
Lymphocytes (×10^9^/L)	1.50 (0.99–1.89)	1.06 (0.70–1.70)	0.07	0.20 (Medium)

*Note:* Values are median (IQR) for continuous variables and number for categorical variables.

^a^ Comparisons made with Mann–Whitney test (continuous variables) or Fisher's exact test (categorical variables); the false discovery rate was controlled by correcting all *p* values using the Benjamini–Hochberg adjustment [22].

^b^ Effect size used for continuous variables was the rank biserial and according to the rules of Funder and Ozer [23], with larger values (positive or negative) indicating larger differences between groups (extremely small: < 0.05: very small: 0.05–0.10; small: 0.10–0.20; medium: 0.20–0.30; large: 0.30–0.40; and very large: > 0.40); effect size used for Fisher's exact test Cramer's *V*, interpreted according to Cohen [24], whereby values of 0.10, 0.30, and 0.50 denoted small, medium, and large effects, respectively.

^c^ Denotes values less or greater than the respective center's laboratory's reference interval.

Neutrophil count on presentation was higher in pugs (14.0 × 10^9^/L; IQR: 9.5–19.8) compared with the dogs of other breeds (10.3 × 10^9^/L; IQR: 7.1–14.4; *p* = 0.02). No difference in lymphocyte count was found between pugs (1.5 × 10^9^/L; IQR: 1.0–1.9) and dogs of other breeds (1.1 × 10^9^/L; IQR: 0.7–1.7; *p* = 0.07).

### 3.3 Histopathological Analysis

Results of histopathological analysis are shown in Table 3. Inflammatory histological patterns included lympho‐plasmacytic, eosinophilic, granulomatous, mixed, neutrophilic, and ulcerative. Neoplastic histological patterns included small‐cell alimentary lymphoma and intermediate to large‐cell alimentary lymphoma. The predominant inflammatory histopathological diagnosis was not significantly different between groups.

**TABLE 3:** Histopathological findings and presence of lymphangiectasia (of any severity) on intestinal biopsy.

Variable	Pugs	Other breeds	*p* ^a^	Effect size^b^
Histology available	32 (68%)	123 (84%)	—	—
Pattern				
Lympho‐plasmacytic	19 (59%)	70 (57%)	1.0	0.00 (Extremely small)
Eosinophilic enteritis	1 (3%)	7 (6%)	1.0	0.00 (Extremely small)
Granulomatous	1 (3%)	0 (0%)	0.98	0.14 (Small)
Neutrophilic	1 (3%)	3 (2%)	1.0	0.00 (Extremely small)
Ulcerative	1 (3%)	3 (2%)	1.0	0.00 (Extremely small)
Mixed	7 (22%)	36 (29%)	1.0	0.00 (Extremely small)
Large‐cell lymphoma	2 (6%)	3 (2%)	0.98	0.03 (Extremely small)
Small‐cell lymphoma	0 (0%)	1 (< 1%)	1.0	0.00 (Extremely small)
Lymphangiectasia	16 (34%)	49 (39%)	0.98	0.00 (Extremely small)

*Note:* Values are median (IQR) for continuous variables and number for categorical variables.

^a^ Comparisons made with Mann–Whitney test (continuous variables) or Fisher's exact test (categorical variables); the false discovery rate was controlled by correcting all *p* values using the Benjamini–Hochberg adjustment [22].

^b^ Effect size used for continuous variables was the rank biserial and according to the rules of Funder and Ozer [23], with larger values (positive or negative) indicating larger differences between groups (extremely small: < 0.05: very small: 0.05–0.10; small: 0.10–0.20; medium: 0.20–0.30; large: 0.30–0.40; and very large: > 0.40); effect size used for Fisher's exact test Cramer's *V*, interpreted according to Cohen [24], whereby values of 0.10, 0.30, and 0.50 denoted small, medium, and large effects, respectively.

### 3.4 Treatment

Treatment recommendations are presented in Table 4. Dietary management included hydrolyzed (e.g., Hypoallergenic or Anallergenic, Royal Canin; z/d, Hill's; HA, Purina; or a recommendation including a combination of any of these diets), highly digestible (Gastrointestinal Low Fat, Royal Canin; i/d, Hill's), low fat (Gastrointestinal Low Fat, Royal Canin; i/d low fat, Hill's), limited ingredient (Sensitivity Duck and Tapioca, Royal Canin; d/d, Hill's; Adult Sensitive Pork and Potato, Burns; Turkey and Sweet Potato, Forthglade; Dermatosis FP, Eukanuba; Duck and Rice; James Wellbeloved; Chappie Original, Mars Petcare; home‐cooked turkey and sweet potato, home‐cooked white fish and potato) and mixed (i.e., more than one diet recommendation) diets. No significant difference was found in the specific dietary recommendation between pugs and dogs of other breeds (*p* = 0.45).

**TABLE 4:** Treatment recommendations between the pug and other‐breed dogs.

	Pugs	Other breeds	*p* ^a^	Effect size^b^
Diet	41 (87%)	132 (89%)	0.45	0.09 (Very small)
Hydrolyzed	29 (71%)	88 (67%)	0.75	0.00 (Extremely small)
Low fat	4 (10%)	9 (7%)	0.40	0.12 (Small)
Other dietary recommendations	8 (20%)	35 (27%)	—	
Immunosuppressive therapy (all)	38 (81%)	117 (79%)	0.75	0.00 (Extremely small)
Corticosteroid only	20 (53%)	68 (58%)	0.75	0.00 (Extremely small)
Other immunosuppressive drugs	18 (47%)	49 (42%)	0.75	0.00 (Extremely small)
Other drugs only	2 (5%)	4 (3%)	—	
Steroids and other drugs	16 (42%)	45 (37%)	—	
Adjunctive treatment				
Antimicrobial	21 (45%)	84 (57%)	0.449	0.07 (Very small)
Fenbendazole	5 (11%)	21 (14%)	0.748	0.00 (Extremely small)
Anti‐thrombotic drug	8 (17%)	32 (22%)	0.748	0.00 (Extremely small)
Cobalamin supplementation	8 (17%)	62 (42%)	0.017	0.21 (Medium)

*Note:* Values are median (IQR) for continuous variables and number for categorical variables. The “Other dietary recommendations” group included highly digestible diets, limited‐ingredient diets, and mixed dietary recommendations. “Single‐agent steroid” refers to animals that were recommended a single‐agent steroid drug at initial presentation. This was compared to a group consisting of: “single‐agent non‐steroidal immunosuppressive drug,” “corticosteroid and an additional non‐steroidal immunosuppressive drug,” and “multiple non‐steroidal immunosuppressive drugs.” This comparison group included multi‐agent chemotherapy (i.e., CHOP), Cyclosporin, Cyclosporin and Dexamethasone, Prednisolone and Azathioprine, Prednisolone and Cyclosporin, and Prednisolone and Chlorambucil.

^a^ Comparisons made with Mann–Whitney test (continuous variables) or Fisher's exact test (categorical variables); the false discovery rate was controlled by correcting all *p* values using the Benjamini–Hochberg adjustment [22].

^b^ Effect size used for continuous variables was the rank biserial and according to the rules of Funder and Ozer [23], with larger values (positive or negative) indicating larger differences between groups (extremely small: < 0.05: very small: 0.05–0.10; small: 0.10–0.20; medium: 0.20–0.30; large: 0.30–0.40; and very large: > 0.40); effect size used for Fisher's exact test Cramer's *V*, interpreted according to Cohen [24], whereby values of 0.10, 0.30, and 0.50 denoted small, medium, and large effects, respectively.

Immunosuppressive treatment was used on initial presentation in 155/195 (79%) dogs, comprising 38/47 (81%) pugs and 117/148 (79%) dogs of other breeds, with no difference in use between groups (*p* = 0.75). For the remaining dogs, either no specific immunosuppressive medication recommendation was made (i.e., diet only), or the dog was euthanized or died at the time of diagnosis. Immunosuppressive agents included prednisolone, chlorambucil, azathioprine, cyclosporine, dexamethasone, multi‐drug chemotherapy protocol (i.e., CHOP), budesonide, and combinations. No differences in use of “single‐agent corticosteroid” vs. “other immunosuppressive drugs” were found between groups (Table 3, *p* = 0.75).

Antimicrobials, anthelmintics, anti‐thrombotics, and cobalamin supplementation were administered to some dogs (Table 3). Fewer pugs (8/47, 17%) received cobalamin than dogs of other breeds (62/148, 42%; *p = 0*.02), but no other significant differences in adjunctive therapy use were identified between groups.

### 3.5 Response to Treatment

No difference in initial response to treatment was found between groups (Chi‐squared test for trend in proportions, *p* = 0.65), with a complete response seen in 12 pugs (29%) and 42 dogs of other breeds (30%), a partial response seen in 18 pugs (43%) and 47 dogs of other breeds (34%), and no response seen in 12 pugs (29%) and 51 dogs of other breeds (36%). However, a between‐group difference in whether dogs responded to any treatment was found (Chi‐squared test for trend in proportions, *p* = 0.02), with a complete response seen in 14 pugs (34%) and 90 dogs of other breeds (64%), a partial response seen in 17 pugs (40%) and 18 dogs of other breeds (13%), and no response seen in 11 pugs (26%) and 32 dogs of other breeds (23%).

### 3.6 Survival

The median duration of follow‐up for the entire data set was 1047 days (IQR: 513–1492). The reason for death or euthanasia was available in 97/116 (84%) dogs, with perceived poor quality of life and lack of response to treatment being reported most frequently (63/95, 66%). Three pugs and four dogs of other breeds died or were euthanized because of suspected pneumonia or aspiration pneumonia. Three pugs and two dogs of other breeds died or were euthanized because of suspected thromboembolic events (4%), whereas two pugs and one dog of another breed suffered cardiopulmonary arrest (3%). Besides these, several other reasons were given but with low numbers for each.

**FIGURE 1:**
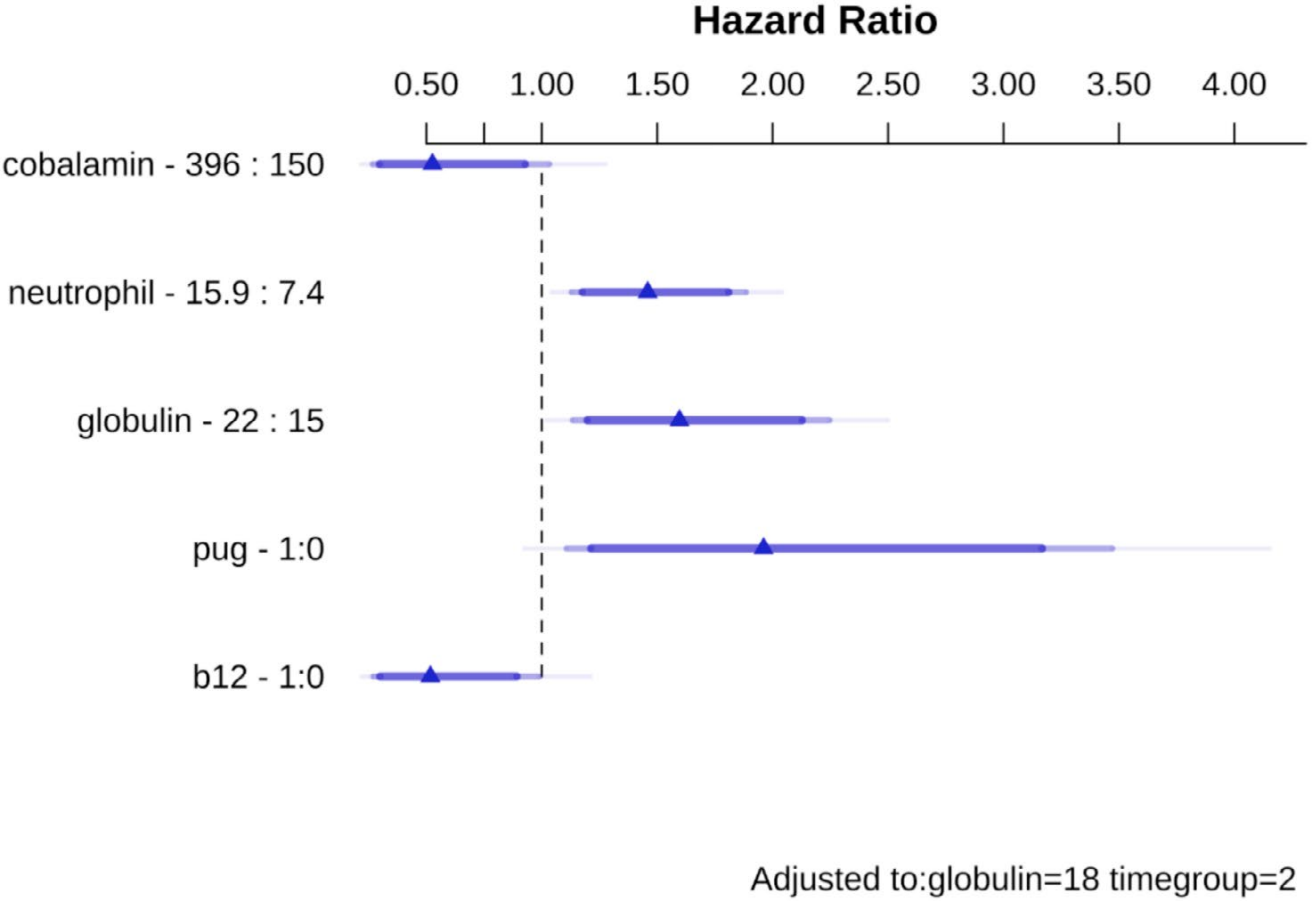
Effects of variables included in the final Cox's proportional hazards regression model. Symbols represent the median hazard ratio for the difference between the 25% and 75% quartiles of each variable. For example, the hazard ratio depicted for neutrophil count is the comparative hazard of having a neutrophil count of 15.9 × 10^9^/L compared to 7.4 × 10^9^/L. The shaded bars display confidence intervals at different levels according to transparency, for example, dark blue (least transparent) 90%, mid blue 95%, and light blue (most transparent) 99%.

Cox's proportional hazards regression was used to determine variables associated with death (death by euthanasia and other causes). On simple regression analysis (Table 5), variables significantly associated with death were age, breed group, increased neutrophil count, serum globulin concentration, and treatment with cobalamin. Although cobalamin concentration (modeled with restricted cubic splines) initially did not meet the criterion for inclusion in the simple regression analysis (linear effect HR: 0.999; 95% CI: 0.996–1.013, *p* = 0.54; nonlinear effect HR: 1.007; 95% CI: 0.992–1.023, *p* = 0.33), it did when included alongside cobalamin supplementation (linear effect HR: 0.999; 95% CI: 0.993–1.000, *p* = 0.07; nonlinear effect HR: 1.018; 95% CI: 1.001–1.035, *p* = 0.04). After refinement, variables that remained in the final model were breed group, neutrophil count, serum globulin concentration, serum cobalamin concentration, and cobalamin supplementation (Figure 1; Table 6).

**TABLE 5:** Simple Cox's proportional hazards regression analysis to determine factors associated with survival.

Variable^a^	Hazard ratio	95% Confidence interval	*p*
Institution			
University of Bristol	Ref	—	—
Davies Veterinary Specialists	0.550	0.127–2.389	0.43
University of Edinburgh	0.484	0.237–0.989	0.05
University of Glasgow	0.931	0.486–1.782	0.83
University of Liverpool	0.977	0.537–1.776	0.94
Northwest Veterinary Specialists	0.869	0.425–1.776	0.70
Willows Veterinary Centre and Referrals	1.092	0.557–2.144	0.80
Age (per year)^b^			
Linear	0.949	0.856–1.053	0.33
Time group 2	1.122	0.960–1.312	0.15
Time group 3	1.369	1.045–1.794	**0.02**
Sex (male vs. female)	0.839	0.573–1.227	0.37
Neuter status (neutered/entire)	1.023	0.647–1.619	0.92
Weight (per kg)	1.002	0.983–1.023	0.77
Pug vs. other‐breed dog	2.080	1.368–3.162	**< 0.001**
Total protein (per g/dL)^b^			
Linear	0.974	0.939–1.010	0.15
Time group 2	1.046	0.997–1.098	0.06
Time group 3	1.053	0.959–1.157	0.28
Albumin (per g/dL)^b^			
Linear	0.941	0.869–1.019	0.14
Time group 2	1.070	0.956–1.198	0.24
Time group 3	1.096	0.883–1.361	0.41
Globulin (per g/dL)^b^			
Linear	0.960	0.910–1.013	0.13
Time group 2	1.111	1.033–1.183	**0.004**
Time group 3	1.132	0.971–1.320	0.11
Cobalamin (per ng/L)^c^			
Linear	0.999	0.996–1.002	0.54
Nonlinear	1.007	0.992–1.023	0.33
Neutrophil count (per 1 × 10^9^/L)	1.037	1.014–1.060	**0.001**
Lymphocytes (per 1 × 10^9^/L)	1.003	0.983–1.023	0.77
Lymphangiectasia	0.796	0.480–1.318	0.38
Diet			
Hydrolyzed diet	Ref	—	—
Low fat	1.728	0.811–3.681	0.16
Other	1.189	0.712–1.984	0.51
Steroids	0.785	0.396–1.556	0.49
Other immunosuppressives	1.562	0.928–2.630	0.09
Antimicrobials	1.019	0.679–1.528	0.93
Anti‐thrombotic medication	1.240	0.762–2.017	0.39
Cobalamin supplementation	0.457	0.291–0.717	**< 0.001**
Fenbendazole	0.582	0.286–1.183	0.14

*Note:* Results reported are hazard ratios, and their 95% confidence interval, from simple Cox's proportional hazards models testing the effect of single predictor variables on survival. Bold values represent significance at the < 0.05 level.

^a^ Besides institution, which was tested on its own, all other variables were stratified by institution.

^b^ The proportional hazards assumption was not met for age, total protein, albumin and globulin; this was addressed by adding a time‐dependent interaction, by stratifying survival time into three groups (time group 1: 0–60 days; time group 2: 61–959 days; time group 3: > 959 days); the results reported in the table are for the overall linear effect as well as differences for time groups (compared with time group 1, as the referent category). Ref: referent category (for categorical variables).

^c^ Including restricted cubic splines for cobalamin ensured a better fit to the data, allowing the association between the predictor and outcome variable also to have a non‐linear component (i.e., depicted as linear and non‐linear components to the hazard).

**TABLE 6:** Final Cox's proportional hazard regression analysis, stratified by institution, to determine factors associated with survival.

Variable	Hazard ratio	95% Confidence interval	*p*
Pug vs. other‐breed dog	1.961	1.108–3.471	**0.02**
Neutrophil count (×10^9^/L)	1.045	1.014–1.077	**0.004**
Globulin (per g/dL)			
Linear	0.949	0.891–1.011	0.11
Time group 2	1.126	1.040–1.219	**0.003**
Time group 3	1.253	1.048–1.497	**0.01**
Cobalamin (per g/dL)			
Linear	0.995	0.991–0.999	**0.05**
Nonlinear	1.020	1.002–1.038	**0.03**
Cobalamin supplementation	0.517	0.271–0.988	**0.05**

*Note:* Results reported are hazard ratios, and their 95% confidence interval, from the best‐fit multiple Cox's proportional hazards model, stratified by institution. The proportional hazards assumption was not met for globulin concentration; this was addressed by adding a time‐dependent interaction, by stratifying survival time into three groups (time group 1: 0–60 days; time group 2: 61–959 days; and time group 3: > 959 days). Bold values represent significance at the < 0.05 level.

**FIGURE 2:**
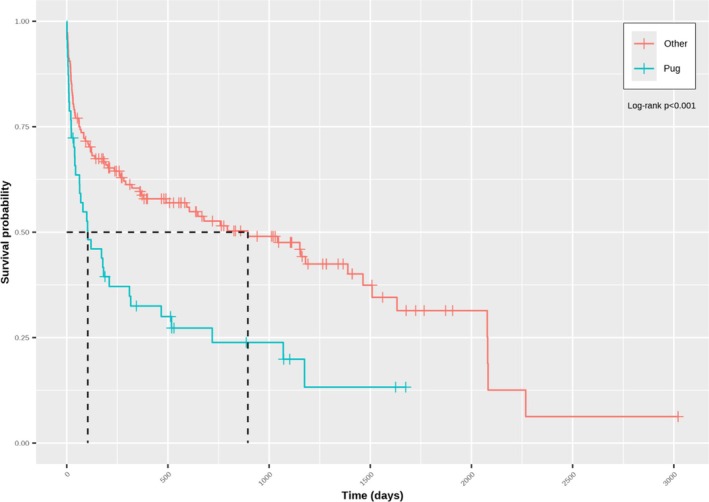
Kaplan–Meier curve depicting survival in pugs (blue) compared to dogs of other breeds (red). Vertical lines represent censored dogs and reflect the time from diagnosis until entry into the data set. Median survival was shorter in pugs compared to dogs of other breeds (*p* < 0.001).

**FIGURE 3:**
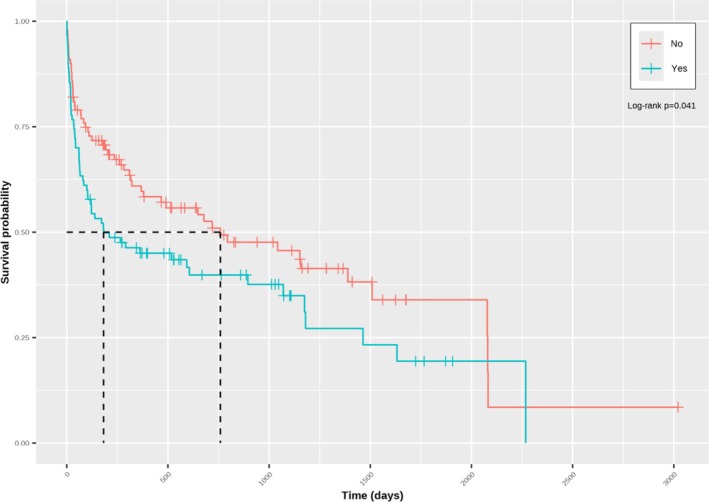
Kaplan–Meier curve depicting survival in dogs with neutrophilia (Yes; black) compared to dogs that did not have neutrophilia (No; gray), as determined by that center's laboratory's reference interval. Vertical lines represent censored dogs and reflect the time from diagnosis until entry into the data set. Median survival was shorter in dogs with neutrophilia compared to those that did not have neutrophilia (*p* = 0.041).

The hazard of death was higher in pugs (HR: 1.961; 95% CI: 1.108–3.741; *p* = 0.002) than in dogs of other breeds, whereas higher neutrophil counts were associated with an increased hazard of death (HR change per 1 × 10^9^/L 1.045; 95% CI: 1.014–1.077; *p* = 0.004). This was reflected in a shorter median survival in pugs (104 days, 95% CI: 63–316) compared with dogs of other breeds (895 days; 95% CI: 491–1509, *p* < 0.001; Figure 2), and a shorter median survival in dogs with neutrophilia (a neutrophil count higher than the RI; 182 days; 95% CI: 103–1070), compared to those that did not have neutrophilia (759 days; 95% CI: 380–2080, *p* = 0.04; Figure 3). Although no significant linear association was found between serum globulin concentration and the hazard of death (HR per g/dL, 0.949; 95% CI: 0.891–1.011; *p* = 0.11), a time‐dependent effect on survival was evident. In this regard, serum globulin concentration was positively associated with the hazard of death in dogs surviving 61–959 days (time group 2: HR: 1.126; 95% CI: 1.040–1.219) and > 959 days (time group 3; 1.253; 95% CI: 1.048–1.497), but not in those surviving 0–60 days (time group 1; HR: 0.949; 95% CI: 0.891–1.011).

**FIGURE 4:**
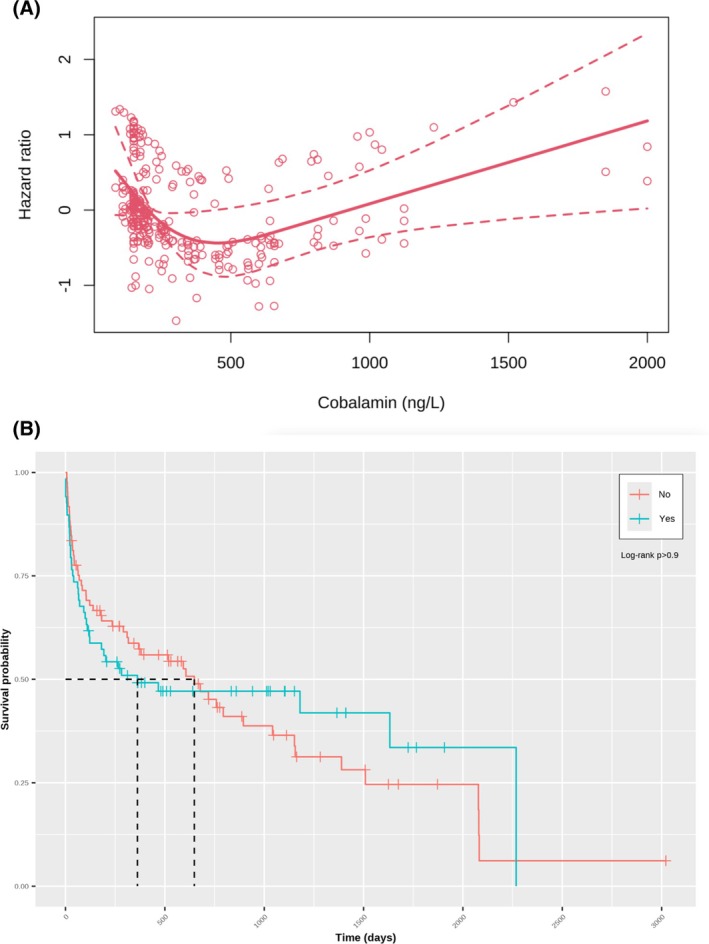
(a) Relationship between cobalamin concentration and hazard ratio in the final multiple regression model. The solid red line represents the median hazard ratio across the range of cobalamin concentrations, dotted red lines indicate the 95%‐confidence intervals, and red circles represent partial residuals from the model. (b) Kaplan–Meier curve depicting survival in dogs with cobalamin less than (blue) compared to greater than (red) the lower limit of respective reference interval. Vertical lines represent censored dogs and reflect the time from diagnosis until entry into the data set. There was no difference in survival between groups (*p* > 0.900).

**FIGURE 5:**
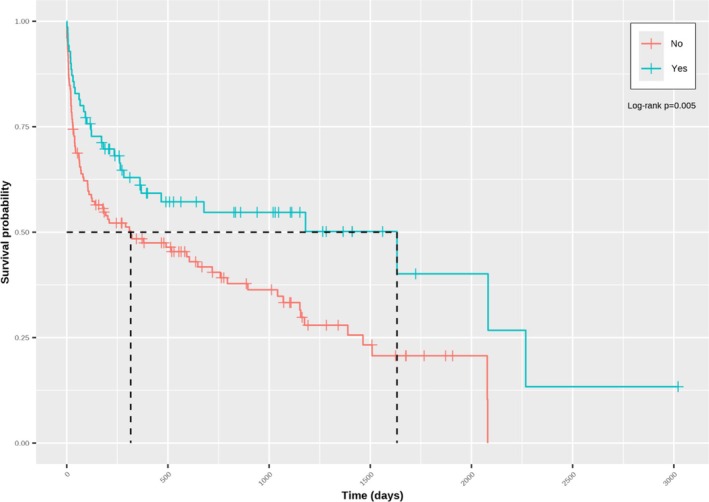
Kaplan–Meier curve depicting survival in dogs that did (blue) and did not (red) receive cobalamin supplementation. Vertical lines represent censored dogs and reflect the time from diagnosis until entry into the data set. Median survival was longer in dogs that received cobalamin supplementation compared to those that did not (*p* = 0.005).

A nonlinear (U‐shaped) association was found between serum cobalamin concentration and hazard of death (Figure 4a), with the highest hazard at lower and higher cobalamin concentrations. Similar findings were evident when the association between survival and serum cobalamin concentrations was assessed with a Kaplan–Meier curve (Figure 4b); in the first approximately 600 days, survival was worse in dogs with subnormal serum cobalamin concentrations, but survival between groups was similar later on, and no overall difference between groups was evident (log‐rank test *p* > 0.9). Finally, the hazard of death was less in dogs that received cobalamin supplementation (HR: 0.517; 95% CI: 0.271–0.988; *p* = 0.05; Figure 5) than in those that did not.

A sensitivity analysis was conducted to determine the effect of missing data on the analysis by repeating the final model on a data set where missing data were replaced using multiple imputation; results were broadly similar (Table S1), with breed (*p* = 0.03), neutrophil count (*p* = 0.01), serum globulin concentration (linear *p* = 0.06; time group 2 *p* ≤ 0.001; and time group 3 *p* = 0.05), and cobalamin supplementation (*p* = 0.02) having significant effects, but the effect of serum cobalamin concentration was no longer significant (linear *p* = 0.76; nonlinear *p* = 0.56).

## 4 Discussion

In our study, a negative association between the pug breed and survival was identified in dogs diagnosed with PLE at UK referral centers. Previous studies have varied greatly in reported median survival, from 30 to 1300 days, suggesting heterogeneity in severity among dogs [1, 2, 3, 4, 25, 26, 27, 28, 29, 30]. The median survival time for pugs in our study (104 days) suggests that the form of this syndrome in pugs is more severe than that seen in dogs of other breeds seen at UK referral hospitals.

Defining response to treatment is complicated, arguably being related both to the resolution of clinical signs and overall survival time. Scoring metrics have been reported for assessing the severity of clinical signs in CE and can be used to monitor response to treatment [23, 31]. Unfortunately, neither CIBDAI nor CCECAI were assessed in our study because they were infrequently used by the attending veterinarians during the study timeframe. Instead, response to treatment was mainly determined by monitoring changes in serum albumin concentration. The use of albumin as a response biomarker can be justified by the fact that it was fundamental to the definition of PLE, is easy to measure, and has been used for the same purpose in previous studies [4, 29, 31]. However, as with survival time, the use of albumin to determine response might not correlate with improvement in clinical signs and quality of life.

In our study, no difference was found between pugs and dogs of other breeds in response to the initial treatment used, but fewer pugs responded completely to any of the treatments used during their care. Although this situation is most likely a consequence of the nature of PLE in pugs, we cannot exclude the possibility of there being differences in treatment recommendations between the groups. That said, other than cobalamin supplementation, no apparent group differences in treatments used were evident between the pug and other breeds. Although this observation may suggest equivalent treatment for both groups, such an assertion cannot be justified given the low numbers of dogs in specific treatment categories. The difference in the use of cobalamin supplementation between groups was most likely to be the result of different serum cobalamin concentrations between groups on presentation, whereby pugs were less likely to present with hypocobalaminemia than were dogs of other breeds. The reason for such a breed difference in cobalamin status is not known but might be related to differences in the region of the small intestine affected, for example, if ileal lesions were less common in pugs.

Previous studies have identified an association between hypocobalaminemia and decreased survival in dogs with PLE [24]. In our study, a “U‐shaped” association between serum cobalamin concentrations and survival was identified, an association that remained after accounting for other variables including breed. As seen in previous studies, the association between hypocobalaminemia and an increased hazard of death was seen [1]. However, an association between hypercobalaminemia and increased hazard of death was seen, although the reasons for this observation are not known. One possibility is the presence of neoplastic disease and, in this regard, an association between increased serum cobalamin concentrations and neoplasia has been reported in cats [22]. That said, to our knowledge, such an association has not yet been identified in dogs. A second possibility is that animals presenting with severe clinical signs may have received cobalamin supplementation before presentation at the referral hospital. Although efforts were made to screen hospital records for such prior cobalamin supplementation, it is possible that it was missed in some cases. In the final model, cobalamin supplementation was independently associated with decreased hazard of death, suggesting a clinical benefit from supplementation in dogs with PLE. However, a randomized controlled trial would be needed to confirm such a clinical benefit.

Our study had some limitations, many of which are related to its retrospective, multicenter design. First, neither the duration nor severity of clinical signs before presentation was assessed, which limits the interpretation of the results. Second, although serum biochemistry results were available for all dogs, such was not the case for other variables including results of histopathological examination, which were not available in 21% of dogs. This difference contributed to a lack of clarity over diagnosis in many cases, a limitation that remains even considering that no association was apparent between survival and whether histopathological analysis had been performed. Although most dogs underwent abdominal imaging as part of their initial investigations, these results were not included as part of the initial data capture. This feature somewhat compounds the issue of lacking some histopathological examination data given that pertinent imaging findings could not be cross‐checked, for example, findings that might be associated with intestinal lymphoma. Third, we decided not to include information about body or muscle condition scores during data collection because these were inconsistently recorded and often utilized different scales, making results difficult to compare. Fourth, we excluded the possibility of protein‐losing nephropathy (PLN) based on decisions of the primary investigators, rather than explicitly requiring that an absence of proteinuria be formally documented (e.g., urine protein:creatinine ratio < 0.5). However, because all cases were seen by a diplomate in either the American or European Colleges of Veterinary Internal Medicine (or by a resident in training supervised by a diplomate), it is unlikely that many cases of PLN were erroneously included. Fifth, laboratory‐specific RIs were used for serum cobalamin concentrations, but it is acknowledged that being within RI serum concentrations might not guarantee adequate cobalamin concentration at a cellular level [32]. Consequently, the difference in cobalamin concentrations documented between groups might not fully reflect differences in cobalamin status.

An additional study limitation was that treatments used were not standardized, resulting in variability of both the initial drug choice and the use of rescue treatment. This lack of standardization likely reflected the absence of a widely accepted standard treatment approach for PLE as well as the fact that opinions on the most effective treatment for PLE likely evolved over the timeframe of the study. Many different immunosuppressive treatments were used, in many different combinations, and this variability was too complicated to enable a meaningful statistical comparison. Similarly, many different diets were used in the study. Given the complexity, we chose to assess two main approaches, the use of a low‐fat diet given that such is recommended for the treatment of lymphangiectasia [1, 33], and the use of a hydrolyzed diets, given their reported treatment advantage in dogs with CE [34]. Although no differences were evident, we cannot exclude a possible role for dietary management in PLE and, in particular, the use of other diet strategies such as single‐source protein diets and home‐prepared diets. A further limitation in assessing response both to drug treatment and diet was the fact that treatment adherence was not assessed. Prospective studies, in particular randomized controlled trials of diet and drug protocols, would be required to determine the most appropriate management for PLE in dogs.

A final study limitation, common to many veterinary studies, was the accurate determination of survival time given that most dogs were euthanized rather than dying of other causes. Decision‐making by owners about euthanasia is complex, with many possible influences, and we cannot be certain that no differences were present between owners of pugs and those of dogs of other breeds. However, given that fewer pugs were euthanized than dogs of other breeds, this factor is unlikely to be the reason for the group differences observed.

## 5 Conclusions

In conclusion, the findings of our study suggest a worse prognosis for pugs with PLE compared with dogs of other breeds diagnosed with PLE at UK referral centers. However, the reasons for this difference are not known, and further research is needed.

## Disclosure

Off‐label antimicrobials may have been used in dogs in this study. Specific names of antimicrobials are not reported in the manuscript (and those used in the United Kingdom generally are licensed under the veterinary cascade).

## Ethics Statement

The University of Liverpool Veterinary Research Ethics Committee reviewed and approved both the animal welfare and research ethics aspects of the project (VREC500ab). Owners gave written permission for anonymized animal data to be included in the research. The authors declare human ethics approval was not needed.

## Conflicts of Interest

Adam G. Gow is currently an employee of Zoetis and holds stock and stock options with Zoetis; however, his work on this project was undertaken at the University of Edinburgh before this employment. Alexander J. German is an employee of the University of Liverpool, but his post is financially supported by Royal Canin, which is owned by Mars Petcare. Alexander J. German also has received financial remuneration for providing educational material, speaking at conferences, and consultancy work for Mars Petcare; all such remuneration has been for projects unrelated to the work reported in this manuscript. The other authors declare no conflicts of interest.

## Supporting information


**Table S1.** Final Cox’s proportional hazard’s regression analysis conducted on a data set where missing values were replaced using multiple imputation, to determine factors associated with survival.
