# Supplementary material for: A Multicenter Observational Study Comparing Survival of Pugs and Dogs of Other Breeds With Protein‐Losing Enteropathy
Source: J Vet Intern Med. 2025 May 15;39(3):e70100. doi: 10.1111/jvim.70100 (PMC12081830; doi:10.1111/jvim.70100)
Supplement: Supplementary file 1 — Table S1. Final Cox’s proportional hazard’s regression analysis conducted on a data set where missing values were replaced using multiple imputation, to determine factors associated with survival. [file JVIM-39-e70100-s001.docx]

**S1 Table.** Final Cox’s proportional hazard’s regression analysis conducted on a dataset where missing values were replaced using multiple imputation, to determine factors associated with survival.

| **Variable** | **Hazard ratio** | | **95% confidence interval** | **P value** |
| --- | --- | --- | --- | --- |
| **Pug vs. other-breed dog** | 1.696 | | 1.057, 2.720 | **0.029** |
| **Neutrophil count** (x10^9^/L) | 1.033 | | 1.009, 1.058 | **0.007** |
| **Globulin** (per g/dL) |  |  | |  |
| Linear | 0.944 | 0.890, 1.001 | | 0.056 |
| Time group 2 | 1.139 | 1.057, 1.226 | | **<0.001** |
| Time group 3 | 1.172 | 1.001, 1.373 | | **0.049** |
| **Cobalamin** (per g/dL) |  |  | |  |
| Linear | 0.999 | 0.996, 1.003 | | 0.761 |
| Non-linear | 1.004 | 0.991, 1.016 | | 0.560 |
| **Cobalamin supplementation** | 0.521 | | 0.308, 0.880 | **0.015** |

Cox’s proportional analysis regression stratified on Institution.
